# Can Coenzyme Q10 Supplementation Reduce Cardiovascular Disease Risk Factors? A Protocol for a GRADE‐Assessed Systematic Review and Dose‐Response Meta‐Analysis of Randomized Controlled Trials

**DOI:** 10.1002/hsr2.70452

**Published:** 2025-02-10

**Authors:** Ali Jafari

**Affiliations:** ^1^ Student Research Committee, Department of Community Nutrition, Faculty of Nutrition Sciences and Food Technology, National Nutrition and Food Technology Research Institute Shahid Beheshti University of Medical Sciences Tehran Iran; ^2^ Systematic Review and Meta‐analysis Expert Group (SRMEG), Universal Scientific Education and Research Network (USERN) Tehran Iran

**Keywords:** cardiovascular diseases, coenzyme Q10, metabolic disease, nutrition, nutritional supplements

## Abstract

**Background and Aims:**

Cardiovascular diseases (CVDs), encompassing a variety of conditions affecting the heart and blood vessels, remain a significant global public health challenge. Among various therapeutic options, Coenzyme Q10 (CoQ10) has garnered attention due to its potential benefits for cardiovascular health. CoQ10 is a naturally occurring antioxidant that plays a crucial role in cellular energy production and has long been used therapeutically. This study aims to summarize and systematically analyze the scientific literature on glycemic profile, lipid profile, anthropometric measures, blood pressure, inflammatory factors, liver function tests, oxidative stress parameters and adipokines associated with CoQ10 supplementation to provide a basis for clinical treatment.

**Methods:**

A systematic electronic search will be conducted to identify articles published from inception to July 2024 across databases including PubMed, Scopus, Web of Science, CENTRAL, and EMBASE. The search will focus on randomized controlled trials (RCTs) involving both healthy and unhealthy participants. Two independent reviewers will evaluate articles, extract data, and assess study quality using the Cochrane risk of bias tool. Discrepancies will be resolved through consultation with a third reviewer. If a sufficient number of eligible studies are identified, a meta‐analysis will be performed on the selected outcomes.

**Results:**

The results will provide a comprehensive synthesis of the effects of CoQ10 supplementation on cardiovascular disease risk factors, including glycemic profile, lipid profile, anthropometric measures, blood pressure, inflammatory factors, liver function tests, oxidative stress parameters, and adipokines.

**Conclusion:**

This protocol outlines a comprehensive approach to systematically review and perform a dose–response meta‐analysis on the effects of CoQ10 supplementation on CVD risk factors. The study will employ rigorous methodologies, including independent evaluation and GRADE assessment, to ensure high‐quality evidence synthesis from RCTs.

## 1 Introduction

Cardiovascular diseases (CVDs) remain a significant global health challenge, causing millions of deaths annually and imposing substantial societal burdens. In 2021, CVDs accounted for 20.5 million deaths worldwide, highlighting their pervasive impact on public health [1]. Despite advances in therapeutic strategies, they continue to be leading causes of mortality globally and in the United States [2]. CVDs, including conditions such as coronary artery disease, heart failure, and hypertension, involve complex pathophysiological mechanisms like inflammation, oxidative stress, and dyslipidemia, collectively posing significant threats to human health [3, 4].

Recent decades have seen increasing attention towards nutritional and pharmacological interventions aimed at reducing CVD risk factors and improving cardiovascular outcomes [5, 6]. Coenzyme Q10 (CoQ10), a vital antioxidant and bioenergetic compound, has garnered substantial interest for its potential cardioprotective effects [7]. CoQ10 plays a pivotal role in cellular energy metabolism, maintaining mitochondrial function, reducing oxidative stress, and modulating inflammation pathways relevant to cardiovascular health [8].

The pathophysiology of CVDs involves intricate interactions among genetic predisposition, lifestyle factors, and environmental influences. This complexity underscores the importance of exploring adjunctive therapies that complement conventional treatments by targeting underlying mechanisms contributing to CVD development and progression [9]. CoQ10 supplementation has emerged as a promising candidate in this regard, with preliminary evidence suggesting beneficial effects on lipid profiles, glycemic control, blood pressure regulation, and markers of inflammation and oxidative stress [10, 11, 12, 13].

While several systematic reviews and meta‐analyses have explored CoQ10 supplementation's effects, they have predominantly focused on isolated outcomes or specific populations, resulting in fragmented and sometimes inconsistent evidence. Previous reviews examined narrow aspects such as liver enzymes (reporting significant reductions in ALT, AST, and GGT levels), athletic performance (focusing on oxidative stress and anaerobic performance), or individual biomarkers of inflammation (showing variable effects on inflammatory markers like CRP, TNF‐α, and IL‐6) [14, 15, 16], Studies in specific populations, such as NAFLD patients, yielded inconsistent results regarding CoQ10's effects on lipid profiles and liver function, with outcomes varying based on dosage and intervention duration [17]. Our systematic review and meta‐analysis protocol addresses these limitations through several innovative methodological approaches that distinguish it from previous research. First, we uniquely combine the grading of recommendations assessment, development and evaluation (GRADE) framework with dose–response meta‐analysis, providing both rigorous evidence quality assessment and practical insights into optimal dosage regimens ‐ a methodological advancement absent in previous reviews. Second, while prior studies typically focused on single outcomes like liver enzymes or specific inflammatory markers, our protocol encompasses a comprehensive range of cardiovascular disease risk factors, including lipid profiles, glycemic control parameters, inflammatory biomarkers, adipokines, oxidative stress markers, anthropometric measures, blood pressure, and liver function. This holistic approach enables a more nuanced understanding of CoQ10's overall impact on cardiovascular health. Third, our protocol employs advanced statistical methods to systematically investigate sources of heterogeneity through detailed subgroup analyses and meta‐regression, addressing a critical gap in previous research where heterogeneity sources were often unexplored or inadequately addressed. Fourth, our methodology includes extensive strategies to minimize publication bias, including comprehensive database searches, inclusion of gray literature, and rigorous statistical assessment of reporting bias ‐ elements often overlooked in previous reviews. Additionally, our protocol's adherence to PRISMA‐P guidelines and PROSPERO registration ensures unprecedented transparency and reproducibility in the review process. This integrated, methodologically robust approach will provide clinicians and policymakers with more comprehensive and actionable evidence regarding CoQ10 supplementation in cardiovascular disease prevention, advancing beyond the limited scope and methodological constraints of previous studies.

Despite the existing literature, a comprehensive systematic review and meta‐analysis integrating high‐quality evidence from randomized controlled trials (RCTs), using the GRADE approach, is warranted. This approach ensures rigorous evaluation of CoQ10's efficacy across diverse populations, encompassing individuals of varying health statuses, ages, and genders. By employing meta‐regression and dose–response analyses, this study aims to provide nuanced insights into the relationship between CoQ10 supplementation dosage, duration, and cardiovascular outcomes, thereby enhancing the precision and applicability of its findings.

In light of these considerations, this protocol outlines a methodologically robust framework for conducting a systematic review and meta‐analysis of RCTs, adhering to international standards of evidence synthesis. By critically appraising available data, our meta‐analysis seeks to advance our understanding of CoQ10 supplementation as a potential adjunctive therapy in the prevention and management of cardiovascular disease, ultimately informing clinical practice and guiding future research endeavors.

### 1.1 Objectives

Our comprehensive meta‐analysis aims to assess the efficacy of CoQ10 supplementation in modifying cardiovascular disease risk factors across both healthy and unhealthy populations. Specifically, we aim to evaluate the impact of CoQ10 on adipokines, anthropometric indices, lipid profiles, blood pressure, inflammation indicators, glycemic control, oxidative stress, and liver function tests, while also investigating heterogeneity across primary studies and other sources.

## 2 Methods

**Figure 1:**
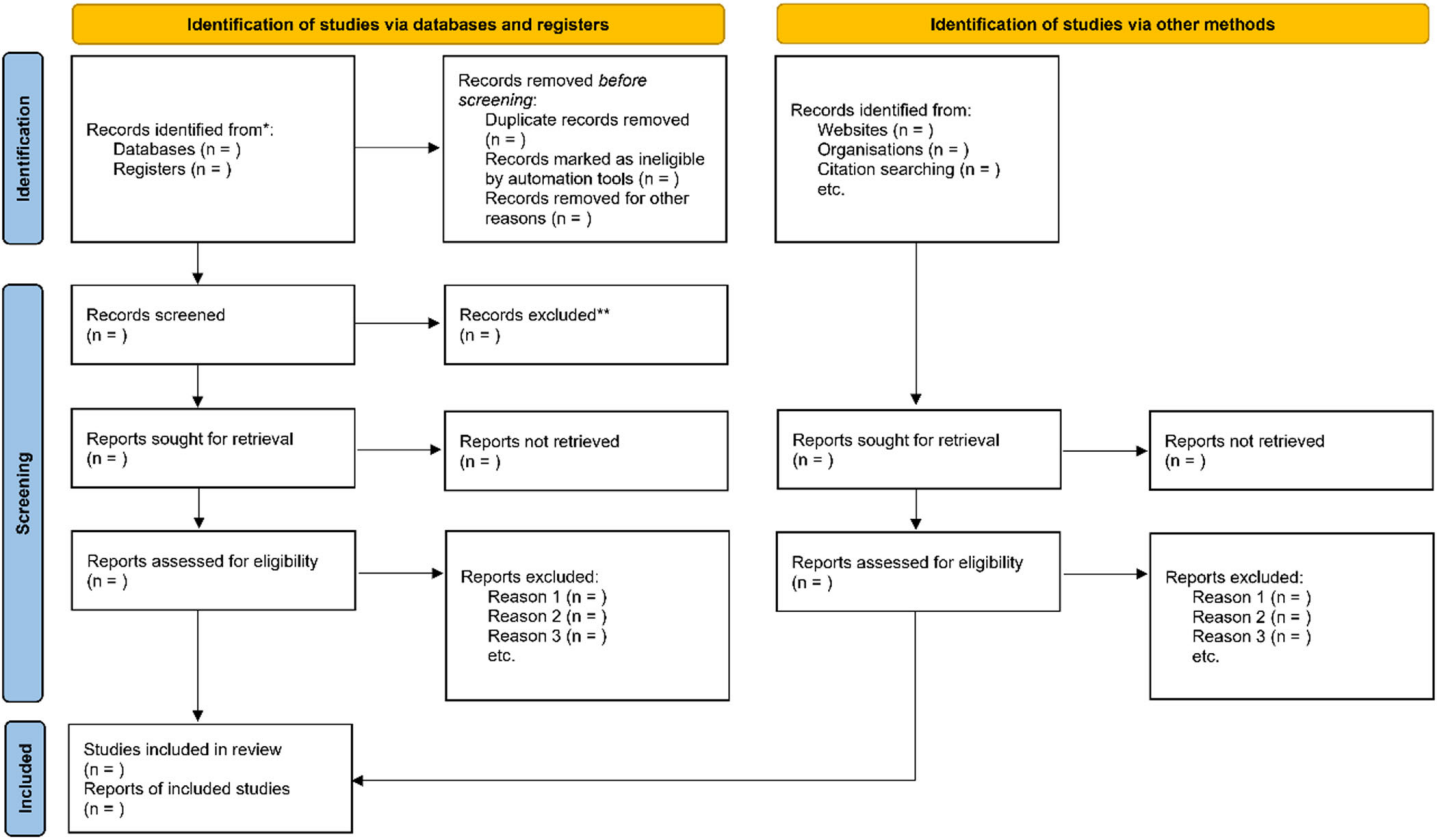
Preferred reporting items for systematic reviews and meta‐analyses (PRISMA) flow diagram of study selection.

The methodology employed in this systematic review aligns with the recommended standards outlined in the 2015 Preferred Reporting Items for Systematic Reviews and Meta‐Analyses (PRISMA) checklist [18]. Additionally, a PRISMA flow chart (Figure 1) will visually represent the quantity of primary studies included or excluded at various stages of this systematic review. The systematic review protocol conforms to the guidelines of Preferred Reporting Items for Systematic Reviews and Meta‐Analyses for Protocols 2015 (PRISMA‐P 2015) [18] and is registered in the international prospective register of systematic reviews (PROSPERO: CRD42024561635).

### 2.1 Eligibility Criteria

#### 2.1.1 Study Design/Characteristics

The systematic review includes primary studies consisting of RCTs with at least single‐blind design and either parallel or crossover designs. Studies must incorporate concurrent control groups and be published in English, spanning from the inception of the field to July 2024. Excluded are non‐RCT methodologies involving human subjects, such as literature reviews, individual case reports, case series, observational studies (including cross‐sectional, case‐control, and cohort studies), and experimental studies conducted on animals or in vitro. Also excluded are conference proceedings, opinion pieces, commentaries, and documents lacking sufficient data on baseline or follow‐up cardiometabolic factors, such as lipid profiles, glycemic management, anthropometric measurements, markers of inflammation, adipose tissue hormones, blood pressure, oxidative stress, and liver function within each study group. Studies discussing coadministration of CoQ10 with other compounds are also omitted.

#### 2.1.2 Subject Types

Inclusion criteria encompass primary research investigating interventions aimed at individuals aged 18 years and older, irrespective of gender.

#### 2.1.3 Intervention(S)

This review focuses on RCTs examining daily supplementation of CoQ10, administered as capsules, tablets, powder, or other forms. Dosage may vary in frequency and quantity, and intervention durations will differ across studies.

#### 2.1.4 Comparator(S)/Control

Studies eligible for inclusion must compare the effects of CoQ10 supplementation with a placebo, no intervention or usual care in a control group.

#### 2.1.5 Outcome(S)

Included studies must report average changes and SDs of inflammatory biomarkers, adipokines, glycemic regulation parameters, anthropometric measurements, blood pressure, liver function markers, and lipid profile components throughout the trial for both intervention and control cohorts, or provide sufficient data to calculate these effect sizes.

#### 2.1.6 Data Sources

The systematic review will systematically search electronic bibliographic databases from inception to July 2024. Specific keywords relevant to CoQ10 will be used in Web of Science, Cochrane Central Register of Controlled Trials, SCOPUS, EMBASE (http://Embase.com), and Medline (http://www.ncbi.nlm.nih.gov/pubmed) (Supporting Information S1: Table S1). Additionally, manual searches of relevant scholarly journals and the references of pertinent articles, meta‐analyses, and review publications will be conducted. Gray literature, including theses and conference abstracts, will also be scrutinized. Contact with study authors via email will be employed to obtain unpublished data, ensuring comprehensiveness.

#### 2.1.7 Search Strategy

The aim of our literature exploration methodology is to identify all relevant RCTs conducted on human subjects. We will employ a carefully selected set of significant search terms to define “CoQ10” and “RCTs.” These keywords are specifically integrated to ensure a comprehensive search that captures all relevant studies, adhering to the principle of search comprehensiveness. Each study will undergo thorough individual assessment during the screening phase, with only those meeting the specified criteria progressing to the analysis phase. A unique search strategy will be tailored for each database, with detailed descriptions of the search approach and syntax used provided in Supporting Information S1: Table S1.

### 2.2 Study Records

#### 2.2.1 Data Management

Two investigators will conduct initial searches of electronic databases following the strategy outlined in the PRISMA‐P statement. Additionally, two researchers will meticulously review the references cited in all included studies. EndNote X7 software will be utilized for data management. The lead reviewer will import search results into an EndNote library and eliminate any duplicate entries.

#### 2.2.2 Selection Process

The selection process for this systematic review will adhere to PRISMA framework guidelines. Identification of relevant studies will involve a three‐step approach. Initially, two reviewers will independently evaluate titles and abstracts of all records identified through database searches, applying specific inclusion/exclusion criteria to identify suitable articles. Any discrepancies during the inclusion process at different stages (title/abstract and full‐text review) will be resolved through consultation with a third researcher specializing in nutrition science and chronic diseases. Subsequently, full texts of potentially eligible articles will be obtained for further assessment. Two reviewers will independently evaluate the full texts of selected abstracts. Detailed documentation of reasons for excluding specific studies will be maintained throughout the selection process and compiled into a table for inclusion in the main article.

#### 2.2.3 Data Extraction

A data extraction form, developed by the lead reviewer and statistician, will be employed to extract data from all selected studies. The team will pilot‐test the data extraction form on five initial studies, making necessary adjustments to ensure reliability. Two reviewers will independently extract information from selected studies. Data extraction will encompass information from published reports or communication with study authors if data in published articles are insufficient. Any discrepancies in the data extraction process that cannot be resolved through discussion will be referred to the project supervisor for resolution.

#### 2.2.4 Data Items

Data retrieval will be organized using the PICOS (Participants, Interventions, Comparisons, Outcomes, Study characteristics) criteria:

a. Study characteristics will include main author identification, research design, study location and duration, country of origin, publication year, and sample size categorized into different groups.
b. Sociodemographic details of participants will encompass age, sex, number of participants, disease type, and initial health status.
c. Interventions and their specific features will include dosage, duration of follow‐up, administration method, and sample size of treatment groups.
d. Outcomes will encompass definitions and measurements of key outcomes related to cardiovascular health, such as inflammatory biomarkers (e.g., CRP, IL‐6), glycemic markers (e.g., FBG, HbA1c), lipid profile components (e.g., LDL‐C, TG), blood pressure, liver function markers, and anthropometric measurements. Variability within primary studies will be evaluated, presenting mean and SD values for cardiovascular risk factors at different time points—preintervention, postintervention, and changes within each group. The analysis will also address other pertinent aspects.

We will synthesize the results for each domain separately to ensure clarity and focus. This approach will emphasize the relevance of each outcome to cardiovascular disease risk and improve the coherence of the findings across diverse outcomes.

### 2.3 Missing Data

Following guidelines established by the Cochrane Institute, our team will initiate correspondence via email with the respective authors of included studies to request additional information if data provided in research reports are deemed insufficient. In cases where initial emails remain unanswered, up to three follow‐up reminders will be sent electronically. If no response is received after these attempts, the absence of requested information will be classified as missing data.

### 2.4 Risk of Bias Assessment

Risk of bias in individual studies will be assessed by two independent reviewers using the Cochrane Collaboration tool [19]. This tool evaluates bias risk across seven domains: sequence generation, allocation concealment, blinding of participants and personnel, blinding of outcome assessment, incomplete outcome data, selective outcome reporting, and other potential sources of bias. Before full application, the tool will be piloted on five primary articles to ensure consistency in assessment. Discrepancies will be resolved by a third reviewer. Bias within each domain will be categorized as low, moderate, or high. The Risk‐of‐bias VISualization (robvis) tool [20] will be utilized to present bias assessments.

### 2.5 Data Synthesis

The software Stata (Version 15.0) (Stata Corp, College Station, Texas) will be employed for the purpose of meta‐analysis. Data will be collected on the means of pre and posttreatment, standard deviations (SDs), and participant numbers in both intervention and placebo groups for various clinical outcomes including lipid profile, glycemic control, anthropometric indices, inflammation, blood pressure, oxidative stress, liver function, and adipokines. The impact of CoQ10 supplementation will be evaluated by analyzing the changes between the intervention and placebo groups. If unit conversion between variables is feasible, the analysis will utilize the weighted mean difference model; otherwise, the standardized mean difference (SMD) model will be applied to account for differences in measurement units. Due to the diversity in populations and settings of the included RCTs, a random effects model will be used to determine the overall effect from the SMDs using the DerSimonian–Laird weighting method. The SDs of the change difference will be calculated using the formula: (SD = square root [(SD pretreatment) 2 + (SD posttreatment) 2 − (2 R × SD pretreatment × SD posttreatment)], with a correlation coefficient (*R*) assumed to be 0.5. The I‐square (*I* ^2^) statistic will be utilized to evaluate between‐study heterogeneity. Statistical heterogeneity will be considered substantial if the *p* value is < 0.05 or if *I* ^2^ value exceeds 50%. Subgroup analyses will be conducted to explore the effects of CoQ10 on clinical outcomes by considering study characteristics like population, follow‐up duration, and CoQ10 dose as potential sources of heterogeneity. Meta‐regression will be used to assess the impact of CoQ10 dosage (g/d) and duration on risk factors related to CVDs. Additionally, a nonlinear model will be applied to combine dose–response data from multiple studies to evaluate the effect of CoQ10 supplementation on CVD risk factors [21]. To examine the influence of individual trials on the meta‐analysis results, the meta‐analysis statistic will be recalculated by omitting one study at a time (leave‐one‐out).

In response to the potential heterogeneity arising from the inclusion of both healthy and unhealthy participants, we will conduct subgroup analyses based on baseline health status. These analyses will differentiate the effects of CoQ10 supplementation on clinical outcomes (e.g., lipid profiles, glycemic control, inflammatory biomarkers) in healthy versus unhealthy populations, allowing for a better understanding of the physiological differences between these groups. Furthermore, we will use meta‐regression techniques to investigate the influence of baseline health status as a covariate, assessing whether variations in population characteristics contribute to heterogeneity in treatment effects.

Sensitivity analyses will be performed to test the robustness of the findings by systematically excluding studies involving specific population subsets, such as those focusing only on healthy or unhealthy participants. This approach will help ensure that the results are not disproportionately influenced by any particular subgroup. All subgroup, meta‐regression, and sensitivity analyses will be transparently reported, with detailed tables and figures illustrating how the effects of CoQ10 supplementation may differ across these populations. This comprehensive approach will enhance the clarity and reliability of our conclusions.

To handle the breadth of outcomes, we will focus on a structured and domain‐specific synthesis of results. This approach ensures that outcomes are presented in a way that aligns with the clinical significance of CoQ10 supplementation, with adjustments made for multiple comparisons and clear reporting of both primary and secondary outcomes.

### 2.6 Assessment of Possible Reporting Bias

An assessment will be conducted to determine the likelihood of bias in reporting outcomes, which encompasses publication bias and other forms of reporting bias. This assessment will involve the use of the counter Funnel plot technique, contingent upon having a sufficient number of studies included in a meta‐analysis (around 10 studies). Begg's test and Egger's test will also be utilized to detect any asymmetry. A significance level of *p* ≤ 0.05 will be employed to indicate a statistically significant association.

### 2.7 Assessing the Quality of the Evidence

GRADE guidelines [22] will assess evidence quality related to CoQ10 effects on cardiovascular disease risk factors. Evaluation will consider study design, bias susceptibility, coherence, relevance, precision, and potential publication bias.

### 2.8 Ethics and Dissemination

No ethical approval is required for this systematic review. Results will be disseminated through peer‐reviewed publication.

### 2.9 Patient and Public Involvement

No patients or public were involved in the design, conduct, or reporting of this research.

## 3 Discussion

This protocol demonstrates several significant strengths that enhance its value and contribution to the field. First, it incorporates advanced methodologies such as the GRADE framework and dose–response meta‐analysis, which are rarely combined in previous reviews. The use of GRADE ensures a systematic evaluation of evidence quality, providing robust and reliable guidance for clinicians and policymakers. Simultaneously, the dose–response meta‐analysis facilitates a deeper understanding of the relationship between CoQ10 supplementation and cardiovascular outcomes, offering practical insights into optimal dosages and intervention durations. These methodological innovations position the study as a benchmark for future research in this domain.

Additionally, the protocol addresses gaps in previous research by encompassing a comprehensive range of cardiovascular disease risk factors, including lipid profiles, glycemic control parameters, inflammatory biomarkers, adipokines, oxidative stress markers, anthropometric measures, blood pressure, and liver function. Unlike prior studies that focused narrowly on select outcomes or populations, this approach allows for a holistic assessment of CoQ10's potential health benefits. The rigorous adherence to PRISMA‐P guidelines, PROSPERO registration, inclusion of gray literature, and strategies to minimize publication bias further reinforce the methodological rigor and transparency of this protocol, ensuring its findings will be both comprehensive and clinically relevant.

Systematic reviews and meta‐analyses are pivotal in synthesizing and summarizing the most robust evidence from healthcare research, effectively bridging the gap between research findings and clinical practice [23]. This study presents a protocol for a systematic review and meta‐analysis aimed at comprehensively evaluating the impact of CoQ10 supplementation on cardiovascular disease risk factors. The findings from this review are anticipated to provide nuanced evidence that can guide healthcare administrators and policymakers in making informed decisions related to cardiovascular disease prevention and public health strategies.

## Author Contributions

**Ali Jafari:** writing – original draft, writing – review and editing, project administration, supervision, data curation, methodology, conceptualization, investigation, validation, software.

## Conflicts of Interest

The authors whose names are listed in this article certify that they have NO affiliations with or involvement in any organization or entity with any financial interest (such as honoraria; educational grants; participation in speakers' bureaus; membership, employment, consultancies, stock ownership, or other equity interest; and expert testimony or patent‐licensing arrangements), or nonfinancial interest (such as personal or professional relationships, affiliations, knowledge or beliefs) in the subject matter or materials discussed in this manuscript.

## Transparency Statement

The lead author Ali Jafari affirms that this manuscript is an honest, accurate, and transparent account of the study being reported; that no important aspects of the study have been omitted; and that any discrepancies from the study as planned (and, if relevant, registered) have been explained.

## Supporting information

Supporting information.

## Data Availability

All relevant data are within the manuscript.
