# Supplementary material for: Can Coenzyme Q10 Supplementation Reduce Cardiovascular Disease Risk Factors? A Protocol for a GRADE‐Assessed Systematic Review and Dose‐Response Meta‐Analysis of Randomized Controlled Trials
Source: Health Sci Rep. 2025 Feb 10;8(2):e70452. doi: 10.1002/hsr2.70452 (PMC11808257; doi:10.1002/hsr2.70452)
Supplement: Supplementary file 1 — Supporting information. [file HSR2-8-e70452-s001.docx]

**Can Coenzyme Q10 Supplementation Reduce Cardiovascular Disease Risk Factors? A Protocol for a GRADE-Assessed Systematic Review and Dose-Response Meta-Analysis of Randomized Controlled Trials**

**Contents**

[**Supplementary Table 1.** Search strategy to find potential eligible randomised controlled trials 2](#_Toc165492129)

| **Supplementary Table 1.** Search strategy to find potential eligible randomised controlled trials |
| --- |

**PubMed**

Limits: -

|  | **Descriptors** |
| --- | --- |
| #1 | ((("Coenzyme Q10"[MeSH Terms]) OR ("Ubiquinone"[MeSH Terms])) OR (("Coenzyme Q10"[Title/Abstract]) OR ("Q10"[Title/Abstract]) OR ("CoQ10"[Title/Abstract]) OR ("Ubiquinone"[Title/Abstract]) OR ("Ubidecarenone"[Title/Abstract]) OR ("Bio-Quinone Q10"[Title/Abstract]) OR ("co-enzyme Q10"[Title/Abstract]) OR ("Ubiquinol-10"[Title/Abstract]) OR ("ubiquinone-10"[Title/Abstract]) OR ("ubiquinone10"[Title/Abstract]) OR ("ubiquinone Q10"[Title/Abstract]) OR ("ubiquinone 50"[Title/Abstract]) OR ("Q-ter"[Title/Abstract]) OR ("ubisemiquinone"[Title/Abstract]))) |
| #2 | Intervention[Title/Abstract] OR "Intervention Study"[Title/Abstract] OR "Intervention Studies"[Title/Abstract] OR "controlled trial"[Title/Abstract] OR random*[Title/Abstract] OR placebo[Title/Abstract] OR "clinical trial"[Title/Abstract] OR Trial[Title/Abstract] OR "randomized controlled trial"[Title/Abstract] OR "randomized clinical trial"[Title/Abstract] OR RCT[Title/Abstract] OR blinded[Title/Abstract] OR "double blind"[Title/Abstract] OR "double blinded"[Title/Abstract] OR trial*[Title/Abstract] OR "Pragmatic Clinical Trial"[Title/Abstract] OR "Cross-Over Studies"[Title/Abstract] OR "Cross-Over"[Title/Abstract] OR "Cross-Over Study"[Title/Abstract] OR parallel[Title/Abstract] OR "parallel study"[Title/Abstract] OR "parallel trial"[Title/Abstract] OR "Clinical Trial" [Publication Type] OR "Randomized Controlled Trial" [Publication Type] OR "Controlled Clinical Trial" [Publication Type] OR "Random Allocation"[Mesh] OR "Randomized Controlled Trials as Topic"[Mesh] OR "Pragmatic Clinical Trial" [Publication Type] OR "Pragmatic Clinical Trials as Topic"[Mesh] OR "Double-Blind Method"[Mesh] OR "Single-Blind Method"[Mesh] OR "Cross-Over Studies"[Mesh] |
| #3 | #1 AND #2 |

**Web of Science**

Limits: -

|  | **Descriptors** |
| --- | --- |
| #1 | TS=("Coenzyme Q10" OR "Q10" OR "CoQ10" OR "Ubiquinone" OR "Ubidecarenone" OR "Bio-Quinone Q10" OR "co-enzyme Q10" OR "Ubiquinol-10" OR "ubiquinone-10" OR "ubiquinone10" OR "ubiquinone Q10" OR "ubiquinone 50" OR "ubisemiquinoneradical" OR "Q-ter" OR "ubisemiquinone") |
| #2 | TS=(Intervention OR “Intervention Study” OR “Intervention Studies” OR “controlled trial” OR random* OR placebo OR “clinical trial” OR Trial OR “randomized controlled trial” OR “randomized clinical trial” OR RCT OR blinded OR “double blind” OR “double blinded” OR trial* OR “Pragmatic Clinical Trial” OR “Cross-Over Studies” OR “Cross-Over” OR “Cross-Over Study” OR parallel OR “parallel study” OR “parallel trial”) |
| #3 | #1 AND #2 |

**Scopus**

Limits: -

|  | **Descriptors** |
| --- | --- |
| #1 | ( TITLE-ABS-KEY ( "Coenzyme Q10" ) OR TITLE-ABS-KEY ( "Q10" ) OR TITLE-ABS-KEY ( "CoQ10" ) OR TITLE-ABS-KEY ( "Ubiquinone" ) OR TITLE-ABS-KEY ( "Ubidecarenone" ) OR TITLE-ABS-KEY ( "Bio-Quinone Q10" ) OR TITLE-ABS-KEY ( "co-enzyme Q10" ) OR TITLE-ABS-KEY ( "Ubiquinol-10" ) OR TITLE-ABS-KEY ( "ubiquinone-10" ) OR TITLE-ABS-KEY ( "ubiquinone10" ) OR TITLE-ABS-KEY ( "ubiquinone Q10" ) OR TITLE-ABS-KEY ( "ubiquinone 50" ) OR TITLE-ABS-KEY ( "ubisemiquinoneradical" ) OR TITLE-ABS-KEY ( "Q-ter" ) OR TITLE-ABS-KEY ( "ubisemiquinone" ) ) OR ( KEY ( "ubiquinone" /exp ) OR KEY ( "coenzyme q10" /exp ) OR KEY ( "ubiquinone" ) OR KEY ( "coenzyme q10" ) OR KEY ( "coq10" ) ) |
| #2 | TITLE-ABS-KEY ( intervention OR "Intervention Study" OR "Intervention Studies" OR "controlled trial" OR random* OR placebo OR "clinical trial" OR trial OR "randomized controlled trial" OR "randomized clinical trial" OR rct OR blinded OR "double blind" OR "double blinded" OR trial* OR "Pragmatic Clinical Trial" OR "Cross-Over Studies" OR "Cross-Over" OR "Cross-Over Study" OR parallel OR "parallel study" OR "parallel trial" ) |
| #3 | #1 AND #2 |

**Embase**

Limits: -

|  | **Descriptors** |
| --- | --- |
| #1 | ('coenzyme q10'/exp OR 'ubiquinone'/exp OR 'coenzyme q10':ti,ab,kw OR 'Q10':ti,ab,kw OR 'CoQ10':ti,ab,kw OR 'ubiquinone':ti,ab,kw OR 'ubidecarenone':ti,ab,kw OR 'Bio-Quinone Q10':ti,ab,kw OR 'co-enzyme Q10':ti,ab,kw OR 'Ubiquinol-10':ti,ab,kw OR 'ubiquinone-10':ti,ab,kw OR 'ubiquinone10':ti,ab,kw OR 'ubiquinone Q10':ti,ab,kw OR 'ubiquinone 50':ti,ab,kw OR 'ubisemiquinoneradical':ti,ab,kw OR 'Q-ter':ti,ab,kw OR 'ubisemiquinone':ti,ab,kw) |
| #2 | 'randomized controlled trial'/exp OR 'randomized controlled trial (topic)'/exp OR 'pragmatic trial'/exp OR 'clinical trial'/exp OR 'clinical trial (topic)'/exp OR 'intervention study'/exp OR 'controlled study'/exp OR 'controlled clinical trial'/exp OR 'double blind procedure'/exp OR 'single blind procedure'/exp OR 'crossover procedure'/exp OR 'parallel design'/exp |
| #3 | #1 AND #2 |

**Cochrane**

Limits: Trial

|  | **Descriptors** |
| --- | --- |
| #1 | ("Coenzyme Q10" OR "Q10" OR "CoQ10" OR "Ubiquinone" OR "Ubidecarenone" OR "Bio-Quinone Q10" OR "co-enzyme Q10" OR "Ubiquinol-10" OR "ubiquinone-10" OR "ubiquinone10" OR "ubiquinone Q10" OR "ubiquinone 50" OR "ubisemiquinoneradical" OR "Q-ter" OR "ubisemiquinone"):ti,ab,kw |
